# A Partition-Based Active Contour Model Incorporating Local Information for Image Segmentation

**DOI:** 10.1155/2014/840305

**Published:** 2014-07-24

**Authors:** Jiao Shi, Jiaji Wu, Anand Paul, Licheng Jiao, Maoguo Gong

**Affiliations:** ^1^Key Laboratory of Intelligent Perception and Image Understanding of Ministry of Education, Institute of Intelligent Information Processing, Xidian University, Xi'an, Shaanxi 710071, China; ^2^School of Computer Science Engineering, Kyungpook National University, Daegu 702-701, Republic of Korea

## Abstract

Active contour models are always designed on the assumption that images are approximated by regions with piecewise-constant intensities. This assumption, however, cannot be satisfied when describing intensity inhomogeneous images which frequently occur in real world images and induced considerable difficulties in image segmentation. A milder assumption that the image is statistically homogeneous within different local regions may better suit real world images. By taking local image information into consideration, an enhanced active contour model is proposed to overcome difficulties caused by intensity inhomogeneity. In addition, according to curve evolution theory, only the region near contour boundaries is supposed to be evolved in each iteration. We try to detect the regions near contour boundaries adaptively for satisfying the requirement of curve evolution theory. In the proposed method, pixels within a selected region near contour boundaries have the opportunity to be updated in each iteration, which enables the contour to be evolved gradually. Experimental results on synthetic and real world images demonstrate the advantages of the proposed model when dealing with intensity inhomogeneity images.

## 1. Introduction

Image segmentation is one of the fundamental tasks in the fields of computer vision and image processing. It has been successfully applied to a variety of realistic problems, such as medical imaging, object recognition, and synthetic aperture radar (SRA) image understanding [1–4]. Currently, various image segmentation techniques have been proposed [5–8], such as histogram thresholding, clustering [7], active contour models [8], and graph-cut methods [5]. However, due to the complexity of several images, designing a robust and efficient segmentation method is still a common goal and challenge for researchers.

Since the introduction of snakes, active contour models (ACMs) [8] have been applied to many fields, in which image segmentation is one of the most important applications [9–14]. The core part of ACMs for image segmentation is that a curve which evolves subject to image characteristics is employed, and then the desired object can be extracted by optimizing an energy function. Nevertheless, the performance of ACMs which use energy functions on the basis of edge information is inadequate, as only objects with edges defined by gradient can be detected [15–18].

Enhanced techniques have been proposed to overcome the limitations of traditional ACMs, especially on designing complex region-based energy functions. Region-based models [19–25] utilize not only the image information near the evolving contour, but also the image statistics information deriving from both sides of the contour. They are less sensitive to noise and more likely to detect weak boundaries when compared to edge-based models. In the Mumford-Shah model [20], an image is decomposed into some regions, and then each region is approximated by a smooth function. However, it is difficult to minimize the function which is not convex in generality. The Chan-Vese (CV) [9] model was proposed based on a region-based energy function inspired by a simplified Mumford-Shah function. The CV model can detect the object with boundaries not necessarily defined by gradient. However, the computational cost of the CV model is rather expensive due to the complicated procedures involved, which limits its application. After the CV model, a broad range of variations have been proposed for reducing computational cost [21].

Traditional ACMs are called piecewise-constant models, since they are designed on the assumption that image intensities are statistically homogeneous of each region over the whole image. However, the assumption is so strict for real world images which are not always statistically homogeneous. In fact, intensity inhomogeneity is frequently observed in real world images and challenging in image segmentation. Due to technical limitations or artifacts introduced by the object being imaged, intensity inhomogeneities often occur in real world images from different modalities and may cause considerable difficulties in image segmentation. For example, intensity inhomogeneity in magnetic resonance (MR) images arises from the nonuniform magnetic fields produced by radio-frequency coils as well as from variations in object susceptibility. It often appears as the variation of intensities from the same tissue type over the locations in an image. In addition, since aurorae are often imaged at night, intensity inhomogeneity in aurora images is usually due to technical limitations of sensor. Segmenting such MR and UVI images has been a challenge. Without an effective preprocessing step such as intensity inhomogeneity correction or histogram equalization, segmentation is difficult to implement.

To deal with intensity inhomogeneity, an effective way is taking local information of the segmented image into account. Because of using local region information, the distance regularized level set evolution (DRLSE) model can cope with intensity inhomogeneity [14]. In addition, some related methods were proposed in [25–27] which have similar capability of handling intensity inhomogeneity as the DRLSE model. However, these local information based methods are sensitive to initial condition to some extent, which holds back their practical applications.

Fuzzy logic which has the ability to flexible process information is widely studied and successfully applied in many real applications [28–30]. The fuzzy logic provides a balanced technique with a strong ability to reject “weak” local minima. Fuzzy energy-based active contour model (FAC) was proposed by Krinidis and Chatzis [31], which first combines fuzzy logic with the active contour methodology. Fuzzy active contour models have a strong ability to reject local minima and can detect objects with smooth or discontinuous boundaries. Besides, to reduce the computational cost, the associated Euler-Lagrange equations were replaced by pseudo-level set functions. Thus, fuzzy active contour models have the properties of less sensitivity to initial conditions and high computation speed. Some related methods which have similar capability of rejecting local minima as the FAC model were proposed in [26, 28, 31].

Although fuzzy active contour models have made great progress, they still have some disadvantages. (1) Traditional fuzzy active contour models fail to process images with intensity inhomogeneity by using distinct means of pixel intensities, one representing the objects region and the other representing the background. (2) The real intention of ACMs is to evolve the region near contour boundaries, which cannot be achieved by the traditional fuzzy active contour models. In fuzzy active contour models, the membership degrees of all the pixels are updated at each iteration, which may cause incorrect results, especially when the contour is not continuous enough [28]. To deal with the difficulties caused by intensity inhomogeneity, a partition-based fuzzy active contour model is proposed for image segmentation. In particular, the following techniques are introduced. (1) By introducing image local characteristics, distinct means of pixel intensities are replaced by spatially varying ones which is better suited for images with intensity inhomogeneity. (2) Regions near contour boundaries are detected with the help of shadowed sets. Only the pixel within the selected region has the opportunity to be updated, which enables the contour to be evolved gradually. The main advantages of the proposed model can be concluded as (1) computational simplicity (the calculation of each step only includes the computation of pixels within the region near the contour boundary); (2) flexibility (it has less sensitivity to initial conditions with the help of fuzzy technique); (3) being suitable for images with intensity inhomogeneity by considering image local characteristics.

The rest of this paper is organized as follows. In Section 2, the main ideas of the proposed model and our motivation will be introduced.  Section 3 will describe the proposed model in detail. In Section 4, experimental results on synthetic images, medical images, and natural images will be described. Conclusions will be drawn in Section 5.

## 2. Motivation

The basic idea of traditional ACMs is to look for the partition of a given image *I* into two regions which have distinct means of pixel intensities, one representing the objects region and the other representing the background. First, a random initial partition of the image is provided. This initial partition defines a curve *C* that will be iteratively evolved according to the image characteristics in the image domain *Ω* by a minimization process. The evolving curve *C* defines a boundary of the segmentation region. The object to be detected is represented by the region inside *C*, while the background is represented by the region outside *C*. In ACMs, the segmentation process is defined as the minimization of the distances between pixels and cluster prototypes, taking into account the length term as a regularization term.

The segmentation result of traditional ACMs is highly dependent on initial conditions. Soft computing technique which has the ability to flexibly process information has been successfully applied in many real applications [28–30], but not in active contour methods. Fuzzy energy-based active contour model (FAC) was proposed by Krinidis and Chatzis [31], which first combines fuzzy logic with the active contour methodology. In the FAC model, the segmentation process is defined as the minimization of a fuzzy energy function [26, 28, 31]:

(1) F(C,c1,c2,u) =μ·Length(C)+λ1∫Ω[u(x,y)]m  ×|I(x,y)−c1|2dx dy+λ2∫Ω[1−u(x,y)]m  ×|I(x,y)−c2|2dx dy,

where *c*_1_ and *c*_2_ are the average intensities of regions inside and outside of the evolving curve *C*, respectively, (*x*, *y*) is the spatial coordinate of a pixel, *u*(*x*, *y*) is the membership degree of the pixel (*x*, *y*) belonging to the inside of *C*, *m* ≥ 1 is the fuzzy coefficient, *I*(*x*, *y*) is the gray value of the pixel (*x*, *y*), and *λ*_1_, *λ*_2_ > 0 and *μ* ≥ 0 are fixed parameters. The first term in (1) is the length term, which accounts for smoothing the curve. *μ* is used to control the effect of the length term. According to [26, 28, 31], the length term is not important for a clean image. For simplicity, without losing the generality, the length term has not been considered during minimizing the fuzzy energy function. *λ*_1_ and *λ*_2_ are used to control the weights of the distances between pixels and average prototypes of the image regions inside and outside *C*, respectively. *λ*_1_ and *λ*_2_ are generally set to 1 for balancing the weights of two regions in (1). *c*_1_ and *c*_2_ are defined as follows:

(2) c1=∫Ω[u(x,y)]mI(x,y)dx dy∫Ω[u(x,y)]mdx dy,c2=∫Ω[1−u(x,y)]mI(x,y)dx dy∫Ω[1−u(x,y)]mdx dy.

Keeping *c*_1_ and *c*_2_ fixed and minimizing (1), the membership degree of each pixel is computed as follows:

(3) $$\begin{array}{c} u\left(x,y\right)=\frac{1}{1+{\left(\lambda_{1}{\left(I\left(x,y\right)-c_{1}\right)}^{2}/\lambda_{2}{\left(I\left(x,y\right)-c_{2}\right)}^{2}\right)}^{1/\left(m-1\right)}}. \end{array}$$

Fuzzy active contour models have a strong ability to reject local minima and can detect objects with smooth or discontinuous boundaries. Besides, to reduce the computational cost, the associated Euler-Lagrange equations were replaced by pseudo-level set functions. Although fuzzy active contour models have made great progress, they still have some difficulties in processing images with intensity inhomogeneous, which will be analyzed as follows.

### 2.1. Motivation of Introducing Local Region Information

ACMs are called piecewise-constant models, since they are designed on the condition that images are approximated by regions with piecewise-constant intensities. It can be seen from (2) that the prototypes are constant, representing the average intensities of pixels within regions inside and outside of the curve, respectively. However, regions are not always statistically homogeneous in real world images. It is inappropriate to employ constant prototypes for describing the image where the intensity distributions overlap between the object region and the background.

**Figure 1:**
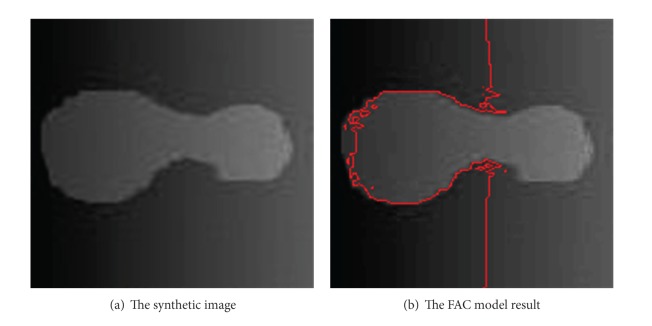
The segmentation result of the FAC model on a synthetic image.

**Figure 2:**
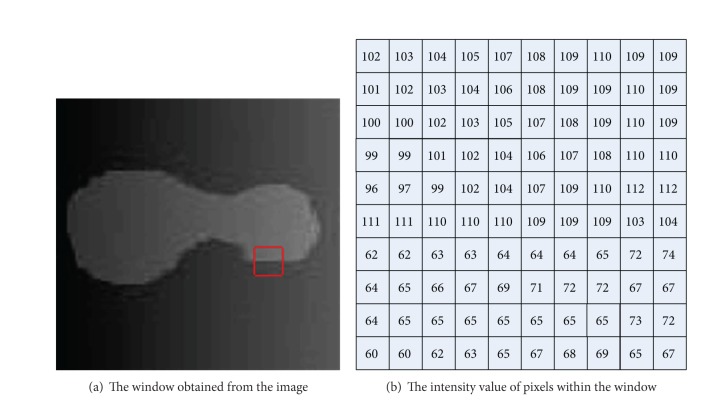
A 10 × 10 window obtained from a synthetic image marked with a red rectangle.

Figure 1 shows the segmentation result of the FAC model on a synthetic image with intensity variation. The segmentation result shows that certain parts of the object with weak intensities are submerged in the background. It is impossible for the FAC model to obtain a satisfactorily result on the synthetic image with intensity variation.  Figure 2(b) depicts a 10 × 10 window shown in a red square extracted from Figure 2(a). The intensity of pixels within this window ranges from 60 to 112. The average intensities of regions inside and outside of the curve obtained by the FAC model are 74.96 and 24.89, respectively. Since the intensities of pixels within this window are more similar to those in the region inside the curve, all the pixels within this window are partitioned into the object region, which will incorrectly partition some pixels within the background into the object region.

Naturally, each pixel has connections with its neighbors to some extent and is reasonable to be approximately described by taking its local immediate neighborhood into consideration. Generally, the local information is an important feature to describe the relationship of pixels within a local region. For a point *x*, its intensity can be approximated by a weighted average of the pixel intensity *I*(*y*) where *y* is the neighborhood of *x*. By introducing image local characteristics, a mild assumption that the image is approximated by regions with piecewise-constant intensities within a local region is more suitable for real world images. In this study, we try to incorporate local region information into the original piecewise-constant models for processing the image with intensity inhomogeneity. The proposed technique is described in Section 3.1.

### 2.2. Motivation of Detecting the Contour Boundary

In fuzzy active contour models, the membership degree measures the degree of a pixel belonging to the object region. The range of the membership degree is [0, 1]. For a binary classification problem, pixels with membership degrees equal to 0.5 form the contour boundary. If the membership degree of a pixel is larger than 0.5, the pixel belongs to the object region. If the membership degree of a pixel is smaller than 0.5, the pixel belongs to the background region. A large uncertainty may exist when assigning pixels with membership degrees near 0.5, because they have nearly the same degrees of belonging to the object region and the background. Thus, it is natural to make great effort to process pixels with large uncertainties within certain selected region of interest.

In addition, ACMs use a high order function to define the contour boundary and originally intend to evolve the region near the contour boundary. However, in traditional fuzzy active contour models, membership degrees of pixels are updated over the whole image domain in each iteration. These approaches fail to only evolve the regions near contour boundaries. Therefore, it is necessary to design a narrow band strategy for making the contour evolve gradually.

To confine the update area close to contour boundaries, it is natural to update the pixel with the membership degree of 0.5. However, it is not easy to accurately define contour boundaries by detecting the pixel with the membership degree of 0.5, because fuzzy energy will create a region with low pixel-density close to the membership degree of 0.5 [28]. An improved model that uses mathematical morphology to detect regions near contour boundaries is stated as follows:

(4) $$\begin{array}{c} u^{\prime }\left(x,y\right)=\frac{\beta \left(H\left(u\left(x,y\right)-0.5\right)\right)⊕R}{1+{\left(\lambda_{1}{\left(I\left(x,y\right)-c_{1}\right)}^{2}/\lambda_{2}{\left(I\left(x,y\right)-c_{2}\right)}^{2}\right)}^{1/\left(m-1\right)}}, \end{array}$$

where *H*(·) is the Heaviside function, *β*(*A*) = *A* − (*A* ⊖ *B*) is a morphological boundary extraction operation that uses a structural element *B* to cause erosion from the image *A*, and *R* is another structuring element for expanding the boundary to its neighborhood. The structuring elements *B* and *R* are a 3 × 3 square and a circle with radius 5, respectively [28]. The selection of structuring elements has a great impact on final segmentation results and requires a careful consideration in practical applications. It is difficult to select a suitable structuring element; different images usually require different structuring elements. According to what has been mentioned above, it is necessary to design a partition-based strategy for making the contour evolve gradually. The designed technique which adaptively detects the region near the contour boundary based on the intrinsic structure of the image is described in Section 3.2.

## 3. Methodology

In this section, an enhanced fuzzy active contour model that draws upon intensity information in local regions is proposed. In particular, the following techniques are designed. (1) The constant prototypes are replaced by the spatially varying ones for overcoming the difficulties caused by intensity inhomogeneities. (2) The region near contour boundaries is automatically detected and only the pixels within the selected region are updated, which enables the contour to evolve gradually. The details are described as follows.

### 3.1. Spatial Varying Prototypes

Traditional ACMs are designed on the assumption that the image is approximated by regions with piecewise-constant intensities over the whole image. However, the assumption is so strict for real world images which are not always statistically homogeneous. A milder assumption that the image is statistically homogeneous within a small local region may better suit real world images. Thus, it is more reasonable to utilize local region information for approximately describing the intensity of a pixel by the weighted average of its neighbors. With the incorporation of local region information, we replace the constants *c*_1_ and *c*_2_ by the spatial varying ones which are computed as follows:

(5) c1(x,y)=∑Ω[u(i,j)]mgk(i,j)I(i,j)∑Ω[u(i,j)]mgk(i,j),c2(x,y)=∑Ω[1−u(i,j)]mgk(i,j)I(i,j)∑Ω[1−u(i,j)]mgk(i,j),

where (*x*, *y*) is the spatial coordinate of the current pixel, (*i*, *j*) is the pixel falling into the local region around the current pixel, *g*_*k*_ is a kernel function which is taken as the weight coefficient assigned to a pixel within the local region around the current pixel ($g_{k}(i,j)=\left(1/\sqrt{2\pi }\sigma \right)e^{-d^{2}/2\sigma^{2}}$with a standard deviation *σ* > 0, and *d* = ((*x* − *i*)^2^ + (*y* − *j*)^2^)^1/2^ is the spatial distance between the pixel (*i*, *j*) and the current pixel (*x*, *y*)). We assume that one pixel is one unit length.

The kernel function is highly useful in clustering analysis and provides a robust property based on influence function analysis [32–35]. It generates larger weight coefficients to pixels closer to the current pixel and smaller weight coefficients to pixels far away from the current pixel. If the spatial distance between pixels (*i*, *j*) and (*x*, *y*) is larger than a level (i.e., (*i*, *j*) keeps away from (*x*, *y*)) [35], the weight coefficient will approach its minimum value. That means the contribution of *I*(*i*, *j*) to *c*_1_(*x*, *y*) and *c*_2_(*x*, *y*) approaches zero as the pixel (*i*, *j*) lies far away from the current pixel. Due to the localization property of the kernel function, the intensity averages of local regions inside and outside of the curve are dominated by the intensities of pixels within the neighborhood of the current pixel [18]. The kernel function makes the influence of pixels within the local region on the current pixel change flexibly according to their spatial distances from the current pixel, allowing more image local characteristics to be obtained. With the incorporation of local region information, the assumption that the image is statistically homogeneous within different small local regions is more appropriate for the characteristics of images with intensity inhomogeneity.

### 3.2. Detecting the Approximation Region Near the Contour Boundary

With the introduction of spatial varying prototypes, the computational cost increases due to the computation of *c*_1_(*x*, *y*) and *c*_2_(*x*, *y*) for each pixel. It is crucial to make great effort to process pixels with a large uncertainty, thus restricting the computational cost to certain selected region of interest. In addition, since pixels with membership degrees near 0.5 form the region near contour boundaries, making great effort to such selected region of interest is benefit to evolve the contour boundary gradually. Thus, we try to confine the update area near the current contour boundary at each iteration, which enables the curve to evolve gradually and saves the computational resource as well.

**Figure 3:**
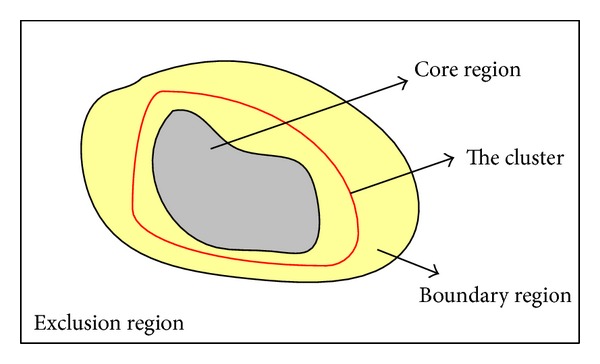
Three levels of belongingness with respect to a cluster.

The concept of shadowed sets was developed to improve the observation and the interpretability of fuzzy sets [36–39]. Shadowed sets help to restrict uncertainty of membership degrees within the whole fuzzy set to certain selected regions to denote patterns with large extent of vagueness and to elevate or reduce membership degrees in other regions. Shadowed sets partition the distribution of a target set into three regions: the core, boundary, and exclusion regions, as shown in Figure 3. The red curve describes the real contour of the cluster. Patterns within the core region belong to the cluster, those within the exclusion region do not belong to the cluster, and those within the boundary region possibly belong to the cluster and come with a certain component of uncertainty. According to shadowed sets theory, great effort will be made to process patterns with large extent of vagueness within the boundary region.

A specific threshold is needed for partitioning the distribution of fuzzy set into three regions. The threshold of object region is computed by minimizing the following function [39]:

(6) V(α)=|ψ1+ψ2+ψ3|,ψ1=∑u(x,y)≤αu(x,y),ψ2=∑u(x,y)≥(Umax⁡−α)(Umax⁡−u(x,y)),ψ3=card(Δ),

where Δ = {(*x*, *y*)∣*α* < *u*(*x*, *y*) < (*U*_max⁡_ − *α*)}, card(Δ) is the number of pixels in Δ, *u*(*x*, *y*) denotes the membership degree of pixel (*x*, *y*) belonging to the object region, *U*_max⁡_ and *U*_min⁡_ are the largest and the smallest membership degrees of all the pixels belonging to the object region, respectively, *ψ*_1_ denotes the reduction of membership degrees, *ψ*_2_ means the elevation of membership degrees, and *ψ*_3_ represents the region with the greatest uncertainty. The three terms on the right side of (6) correspond to the three regions shown in Figure 3 [39]. The optimal threshold can be obtained by satisfying the requirement *α*_opt_ = arg min_*α*_ *V*(*α*). The values of *α* are suggested in the range of [*U*_min⁡_, (*U*_max⁡_ + *U*_min⁡_)/2] with an interval of 0.001 [39].

The membership degrees of pixels belonging to a certain region can be considered as a fuzzy set. After obtaining the thresholds of the object region and the background, the approximate region near the contour boundary can be detected according to the following functions:

(7) Ra=S1∪S2,S1={(x,y) ∣ α1opt<u(x,y)<(max⁡(u(x,y))−α1opt)},S2={(x,y) ∣ α2opt<(1−u(x,y))   <(max⁡(1−u(x,y))−α2opt)},

where *α*_1opt_ and *α*_2opt_ are the adaptive thresholds of the object region and background and are updated in each iteration and *S*_1_ and *S*_2_ are boundary regions inside and outside of the evolving curve, respectively. *R*_*a*_ is the approximation region near the contour boundary, which is adaptively detected with the help of shadowed sets theory.

**Figure 4:**
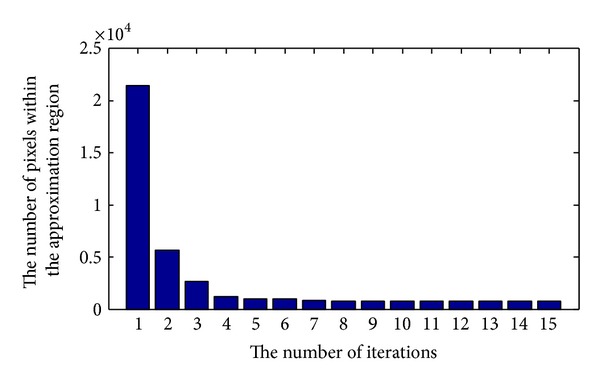
The number of pixels within approximation regions near the contour boundaries changes over time.

**Figure 5:**
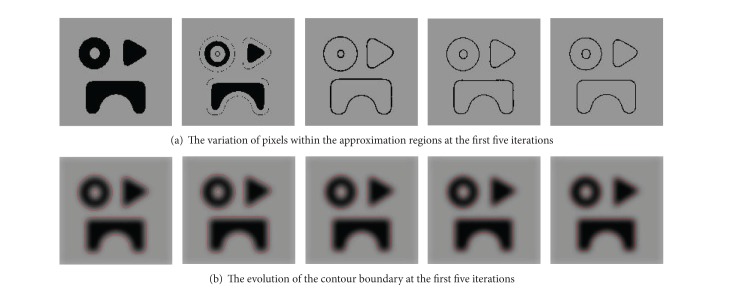
The evolution of the approximation region and the associated contour boundary with iteration.

To demonstrate the effectiveness of detecting the approximation region near the contour boundary, a synthetic image with size 143 × 150 is employed. The variation in the number of pixels within the approximation region is depicted in Figure 4. It can be seen that the number of pixels within the approximate region becomes smaller with time increasing.  Figure 5(a) shows the variation of the approximate region, in which pixels within the approximation region are shown in black and other pixels are shown in gray. The evolution of the corresponding contour boundary (red curve) is depicted in Figure 5(b). It can be found that pixels within the region near the contour boundary are effectively identified, and the approximate region can more accurately approach to the real contour of objects with time increasing.

### 3.3. General Framework of the Proposed Model

We present a partition-based fuzzy active contour model with incorporating local information, termed as LFAC for short. The proposed model designs an enhanced fuzzy energy function which is written in the summation sign form for indicating the discrete nature of the image data. The fuzzy energy function is introduced as follows:

(8) F=∑Ω(u(x,y))m(I(x,y)−c1(x,y))2 +∑Ω(1−u(x,y))m(I(x,y)−c2(x,y))2,

where *c*_1_(*x*, *y*) and *c*_2_(*x*, *y*) are the spatial varying prototypes of the pixel (*x*, *y*), *Ω* denotes the image domain, *u*(*x*, *y*) is the membership degree of the pixel (*x*, *y*) belonging to the object region, *I*(*x*, *y*) is the gray value of the pixel (*x*, *y*), and *m* ≥ 1 is the fuzzy coefficient. The main steps of the proposed model are presented as follows.

**Step 1 .**

Set fuzzy coefficient *m* and the stopping condition *ε* = 10^−2^.

**Step 2 .**

Provide an initial partition of the image; set *u* > 0.5 for the object region and *u* < 0.5 for the background.

**Step 3 .**

Detect the approximation region close to contour boundaries according to (7).

**Step 4 .**

Compute the spatial varying prototypes for each pixel within the approximation region according to (5).

**Step 5 .**

Assume that the membership degree of the current pixel is *u*_*o*_. Compute the new membership degree *u*_*n*_ for the pixel according to (3). Then, compute the difference between the old and the new energy Δ*F* which is derived in the Appendix:

(9) ΔF=(unm(s1s1+unm−uom)2−uom)(Io−c1(x,y))2 +((1−un)m(s2s2+(1−un)m−(1−uo)m)2   −(1−uo)m)(Io−c2(x,y))2,

where *s*_1_ = ∑_*Ω*_[*u*(*i*,*j*)]^*m*^ *g*_*k*_ and *s*_2_ = ∑_*Ω*_[1−*u*(*i*,*j*)]^*m*^ *g*_*k*_. If Δ*F* < 0, *u*_*o*_ is replaced by *u*_*n*_ and vice versa.

**Step 6 .**

Compute the total energy *F* according to (8). If the change of *F* is smaller than *ε*, stop. Otherwise, return to Step 3.

## 4. Experimental Results

In order to assess the effectiveness of the proposed method, both synthetic and real world images which are widely used in the field of image segmentation algorithms are used in the experiments [14, 18, 25, 26, 31]. We compare the proposed model with state-of-art models including the CV model [9], the Geodesic-Aided C-V method (GACV) [6], the distance regularized level set evolution model (DRLSE) [14], and the FAC model [31]. The initial contours are set to be the same for all the compared models in Sections 4.2 and 4.3.

### 4.1. Measuring Segmentation Accuracy

In our experimental study, the results are exhibited in two ways: the final segmentation results in figure form and the criteria in tabular form. Since the ground truth of the synthetic images can be obtained, the performances of the compared models were compared with respect to segmentation accuracy. To evaluate segmentation quality, a region-based segmentation accuracy measurement *P*_mp_ is employed [40]. *P*_mp_ which measures the variation of the extracted region from the desired region is the percentage of mislabelled pixels and is defined as

(10) $$\begin{array}{c} P_{\text{mp}}=\frac{\left|R_{\text{mb}}\right|+\left|R_{\text{ms}}\right|}{\left|R_{b}\right|+\left|R_{s}\right|}\times 100\%, \end{array}$$

where *R*_*b*_ and *R*_*s*_ are the sets of pixels within the desired and extracted regions, respectively, *x* is the pixel in the image, *R*_mb_ = {*x* : *x* ∈ *R*_*b*_∧*x* ∉ *R*_*s*_} is the set of pixels in *R*_*b*_, but not in *R*_*s*_, and *R*_ms_ = {*x* : *x* ∉ *R*_*b*_∧*x* ∈ *R*_*s*_} is the set of pixels in *R*_*s*_ but not in *R*_*b*_. The smaller *P*_mp_ is the better the result is assessed.

### 4.2. Results on Synthetic Images

**Figure 6:**
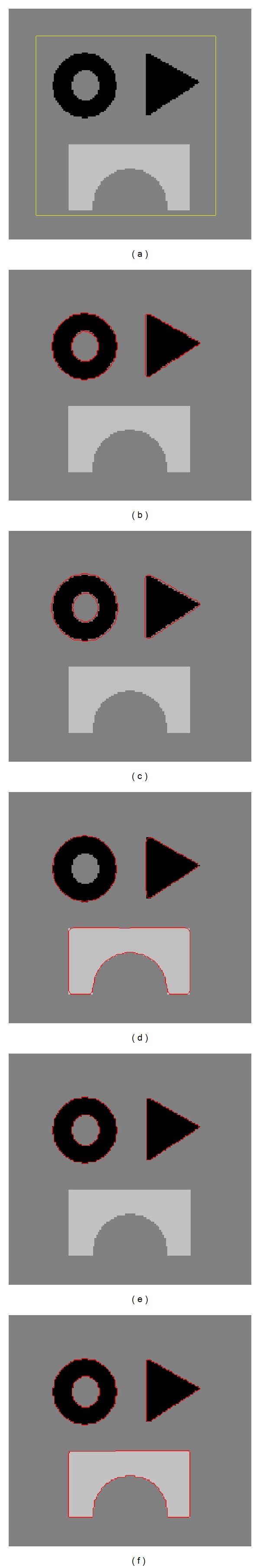
Segmentation results on a synthetic image: (a) the initial contour, (b) the CV model result, (c) the GACV model result, (d) the DRLSE model result, (e) the FAC model result, and (f) the LFAC model result.

The first experiment applies the compared models on synthetic images with different characteristics, in which the intensities of the object regions to be detected are varying.  Figure 6 illustrates that the CV model, the GACV model, and the FAC model fail to detect the object at the bottom, while the proposed model and the DRLSE model succeed. The curve obtained by the CV model or the FAC model better depicts the real contour than that of the GACV model. The DRLSE model can detect the object at the bottom, but the region inside the ring cannot be segmented. As the fact that the intensity of the arch is more similar to that of background, the arch is easily submerged into background when adopting constant prototypes as in the CV model, the GACV model, and the FAC model. The comparison between Figures 6(f) and 6(e) demonstrates that the proposed model detects all the objects by introducing the spatial varying prototypes.

**Figure 7:**
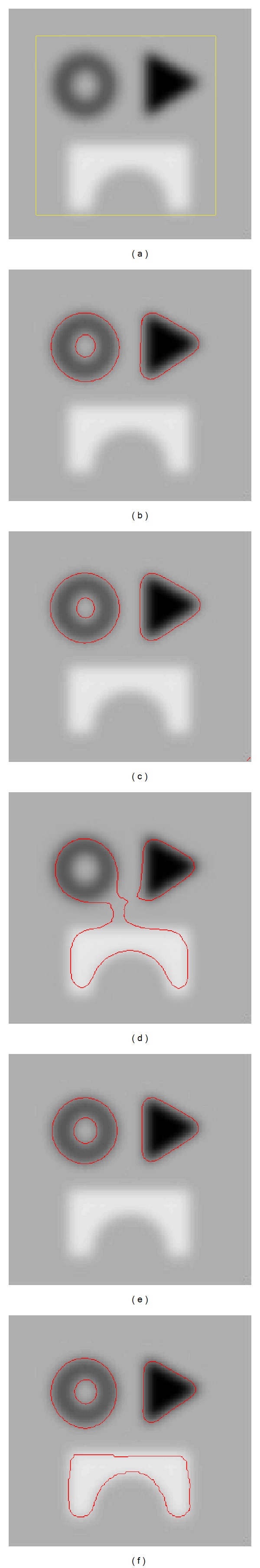
Segmentation results on a synthetic image: (a) the initial contour, (b) the CV model result, (c) the GACV model result, (d) the DRLSE model result, (e) the FAC model result, and (f) the LFAC model result.

We next consider a synthetic image with blurred boundaries as shown in Figure 7(a). Figures 7(b), 7(c), and 7(e) show that similar segmentation results are obtained by the CV model, the GACV model, and the FAC model. The object at the bottom cannot be easily detected by the three models. The curve obtained by the FAC model can better match the real contour inside the ring compared with the CV model and the GACV model. Although the DRLSE model detects a portion of the object at the bottom as shown in Figure 7(d), its segmentation result is not satisfied.  Figure 7(f) shows that the contour boundary of the proposed model can be better tailored to the real contour of three objects.

**Figure 8:**
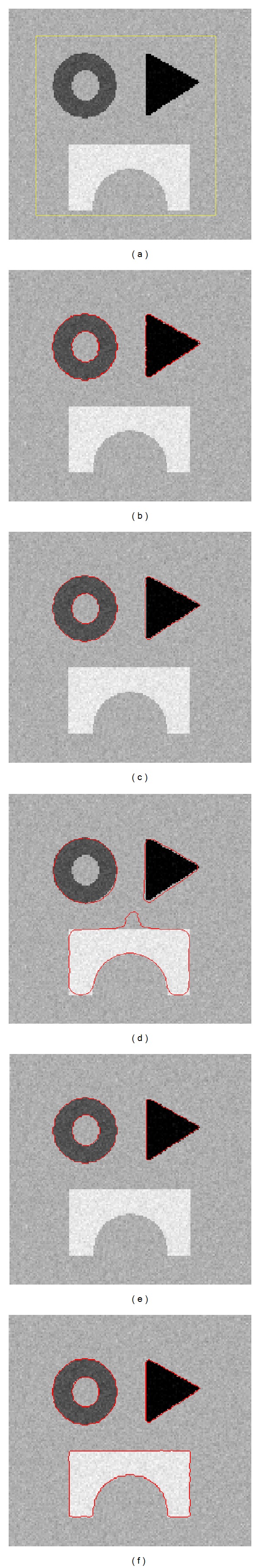
Segmentation results on the synthetic image corrupted by Gaussian noise: (a) the initial contour, (b) the CV model result, (c) the GACV model result, (d) the DRLSE model result, (e) the FAC model result, and (f) the LFAC model result.

**Figure 9:**
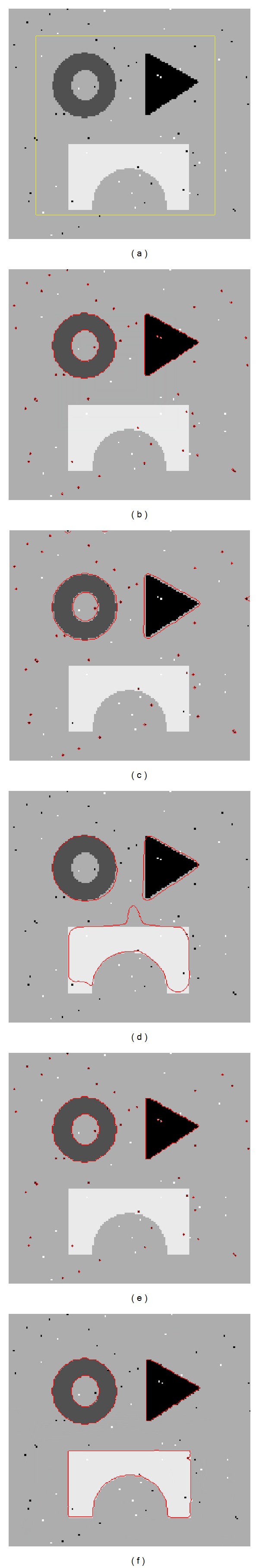
Segmentation results on the synthetic image corrupted by salt and pepper noise: (a) the initial contour, (b) the CV model result, (c) the GACV model result, (d) the DRLSE model result, (e) the FAC model result, and (f) the LFAC model result.

Furthermore, the performance of the compared models under noise conditions is investigated. A synthetic image is corrupted by Gaussian noise and salt and pepper noise. Segmentation results are shown in Figures 8 and 9, respectively. Figures 8(b), 8(c), and 8(e) show that the CV model, the GACV model, and the FAC model are not affected by Gaussian noise, but the object at the bottom cannot be detected by the three models.  Figure 8(d) shows that the DRLSE model detects a large proportion of the object at the bottom. But its result is not satisfactory enough, as the contour inside the ring is unable to be detected.  Figure 8(f) shows that the proposed model removes the added noise and well detects the object contour.

Figures 9(b), 9(c), and 9(e) show that the results obtained by the CV model, the GACV model, and the FAC model are affected by salt and pepper noise to some extent. The result obtained by the CV model is the most seriously affected by noise. The comparison between Figures 9(e) and 9(f) shows that the proposed model performs better than the FAC model. With the accurate estimation of the relationship among neighbors by employing the local region information, the proposed model obtains better performance than the original FAC model.  Figure 9(d) shows that the DRLSE model removes a large proportion of the noise and keeps clear contour boundaries. However, it fails to detect the region inside the ring.  Figure 9(f) shows that the proposed model not only obtains a desire partition but also becomes insensitive to noise.

Table 1 shows the segmentation accuracy in terms of *P*_mp_ obtained by each model. Apart from the proposed model, the DRLSE model obtains the lowest *P*_mp_ on four images among the remaining models. The FAC model performs slightly better than the CV model and the GACV model in terms of *P*_mp_. The value of *P*_mp_ obtained by the proposed model is lower than that obtained by the FAC model. The comparison between the FAC model and the proposed model demonstrates that object regions which are not distinct from the background can be detected to a larger extent by the proposed model. The proposed model obtains the lowest *P*_mp_ on four images, which indicates that the object region detected by the proposed model is more similar to the actual object region compared with those of other four models.

**Figure 10:**
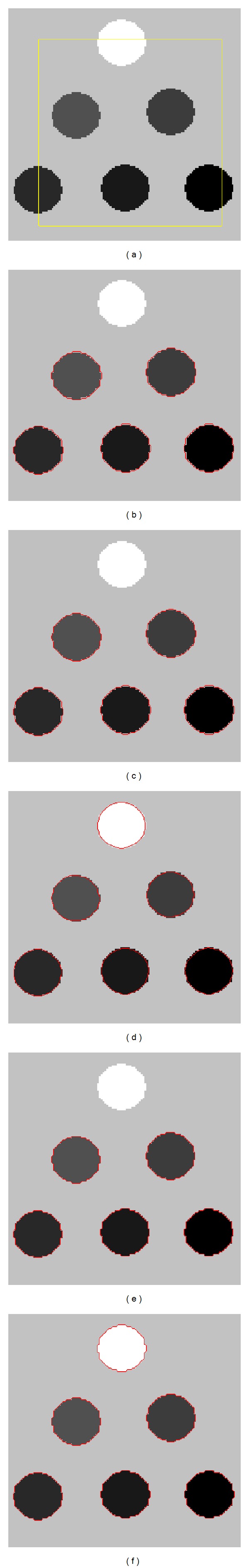
Segmentation results on the synthetic image: (a) the initial contour, (b) CV result, (c) GACV result, (d) DRLSE result, (e) FAC result, and (f) LFAC result.

**Figure 11:**
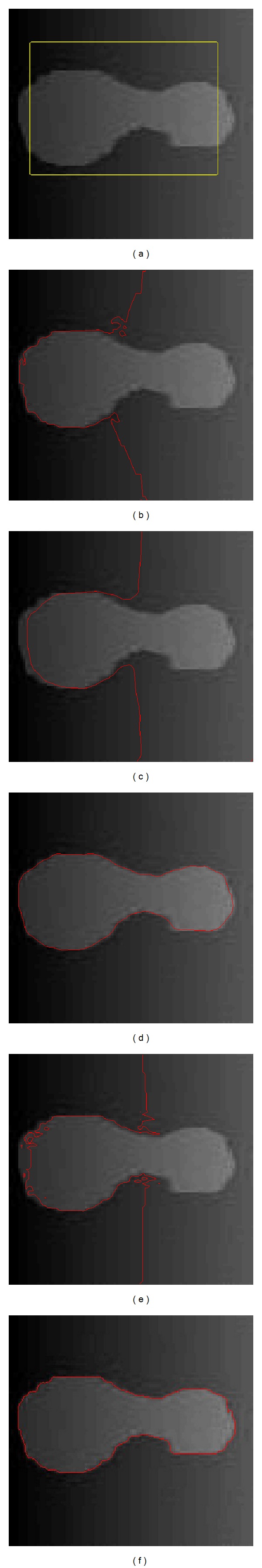
Segmentation results on the synthetic image: (a) the initial contour, (b) CV result, (c) GACV result, (d) DRLSE result, (e) FAC result, and (f) LFAC result.

Moreover, we apply the compared models on other two synthetic images. Since the ground truth of the two synthetic images is not given, only the segmentation result is shown. Figures 10(b), 10(c), and 10(e) show that the CV model, the GACV model, and the FAC model fail to obtain the right segmentation results, as the top dot cannot be detected. Visually, the DRLSE model and the proposed model are able to segment the dot in white as shown in Figures 10(f) and 10(d). The contour boundary of the proposed model can be better tailored to the real contour of objects than that of the DRLSE model.  Figure 11 shows the segment results on a synthetic image where the pixel intensities of background and the object to be detected present high variation. Since, the right part of the background has higher intensities than the left part of object, the FAC model, the CV model, and the GACV model cannot detect the contour of the object at the right side as shown in Figures 11(b), 11(c), and 11(e). Figures 11(d) and 11(f) show that DRLSE and LFAC can better detect the object than other three models.

**Figure 12:**
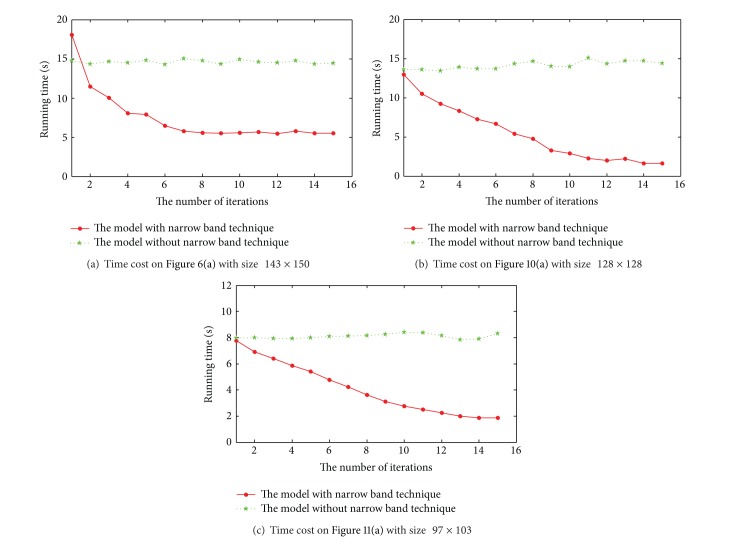
Time cost of the model with and without partition-based technique on three images.

Finally, Figure 12 illustrates the computational cost on three synthetic images used in this subsection with different sizes for the proposed model with and without partition-based technique. All experiments performed on a Pentium IV (3 GHz) workstation under Windows XP Professional using MATLAB. The model without using partition-based technique is computational consuming, since all the pixels are updated in each iteration. With the help of shadowed sets, only the pixels within the boundary region are updated in each iteration. Since the number of pixels within the region close to the contour boundary becomes smaller with time increasing, the time cost of the proposed model employing partition-based technique is less than the model without using partition-based technique as the result shown in Figure 12.

### 4.3. Results on Real World Images

**Figure 13:**
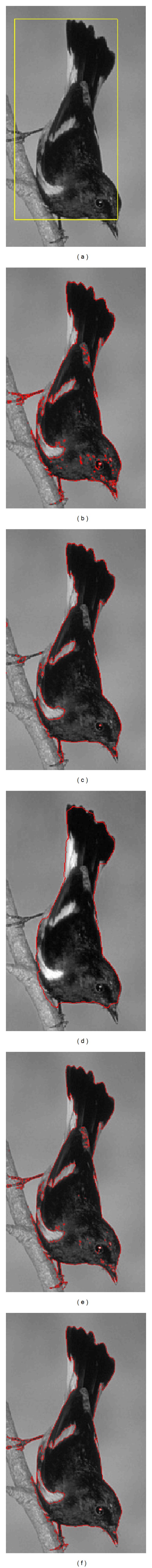
Segmentation results on the bird image: (a) the initial contour, (b) the CV model result, (c) the GACV model result, (d) the DRLSE model result, (e) the FAC model result, and (f) the LFAC model result.

In this part, two medical images and two natural images which are widely used in the field of image segmentation algorithms are used in the experiments [14, 18, 25, 26, 31].  Figure 13 shows that the five models achieve the similar results in general, except in the area near the tree branch and the head of the bird.  Figure 13(b) shows that the CV model fails to detect the head and the claw. Two paws cannot be detected by the GACV model as shown in Figure 13(c).  Figure 13(e) illustrates that the FAC model better detects the paws than the CV model and the GACV model. As shown in Figure 13(d), the DRLSE model achieves a clear contour, while the contour cannot depict real boundaries of the paws and the beak. The proposed model correctly detects the border between the bird and the tree branch. The final curves obtained by LFAC well approach the real contour of the bird, which can be seen from the tail, the paws, and the beak.

**Figure 14:**
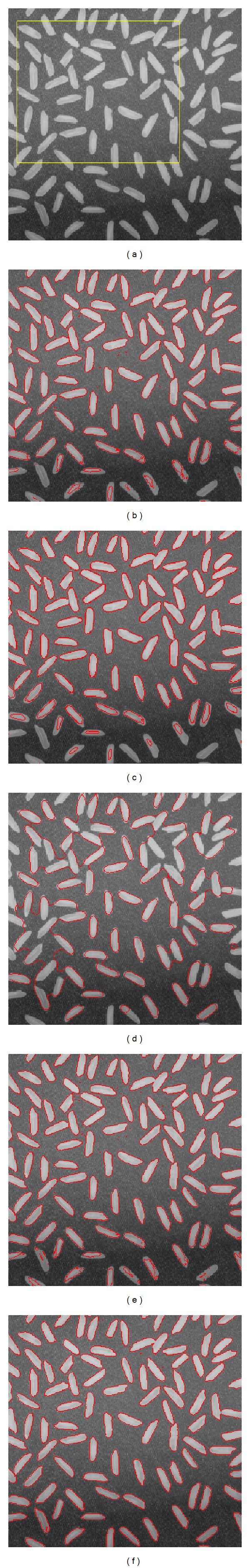
Segmentation results on the rice image: (a) the initial contour, (b) the CV model result, (c) the GACV model result, (d) the DRLSE model result, (e) the FAC model result, and (f) the LFAC model result.

Figure 14(d) shows that the DRLSE model fails to segment the rice image. Figures 14(b), 14(c), and 14(e) depict that the CV model, the GACV model, and the FAC model are unable to correctly detect the real contour of all the rice grains, especially the rice grains at the bottom of the image. Besides, the results obtained by the CV model and the FAC model have some noises on the top of the image. The proposed model extracts the desired contour of all the rice grains as shown in Figure 14(f). With the help of spatial varying prototypes, even the rice grains at the bottom with little difference in terms of the intensity from the background can be well detected.

**Figure 15:**
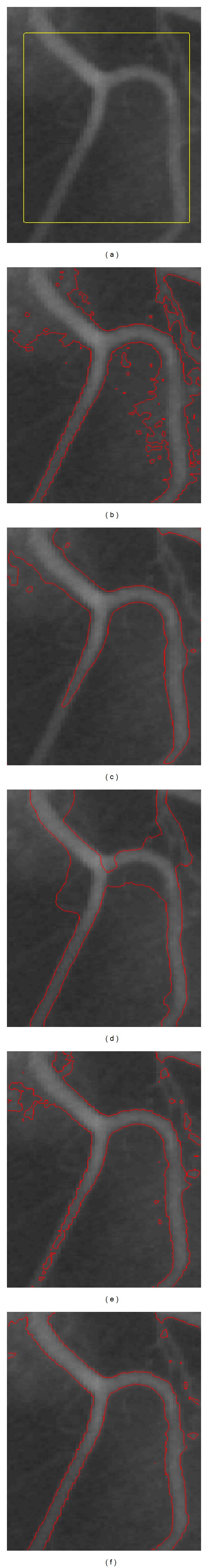
Segmentation results on the first vessel image: (a) the initial contour, (b) the CV model result, (c) the GACV model result, (d) the DRLSE model result, (e) the FAC model result, and (f) the LFAC model result.

**Figure 16:**
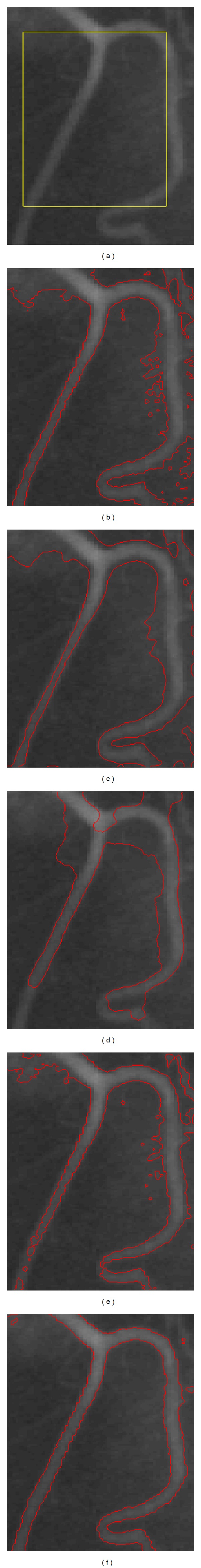
Segmentation results on the second vessel image: (a) the initial contour, (b) the CV model result, (c) the GACV model result, (d) the DRLSE model result, (e) the FAC model result, and (f) the LFAC model result.

Furthermore, the performance of the compared models on two vessel images is evaluated.  Figure 15 shows that the results obtained by the GACV model and the FAC model cannot detect the whole vessel, as the vessel at the bottom left of the image is submerged into the background. Although the results obtained by the CV model and the DRLSE model detect the vessel at the bottom, they fail to approach the real contour of the vessel as shown in Figures 15(f) and 15(e).  Figure 15(f) indicates that that the final contour obtained by the proposed model well depicts the real border of the vessel. Figures 16(d) and 16(e) demonstrate that the results obtained by DRLSE and FAC cannot detect the vessel at the bottom left of the image. Although the segmentation results obtained by CV and GACV detect the vessel at the bottom of the image, they still fail to depict the real contour of the vessel on the right side. The vessel is well detected by the proposed model as shown in Figure 16(f). Moreover, the curve obtained by the proposed model well approach the real border of the vessel.

### 4.4. The Effect of Different Initial Conditions

**Figure 17:**
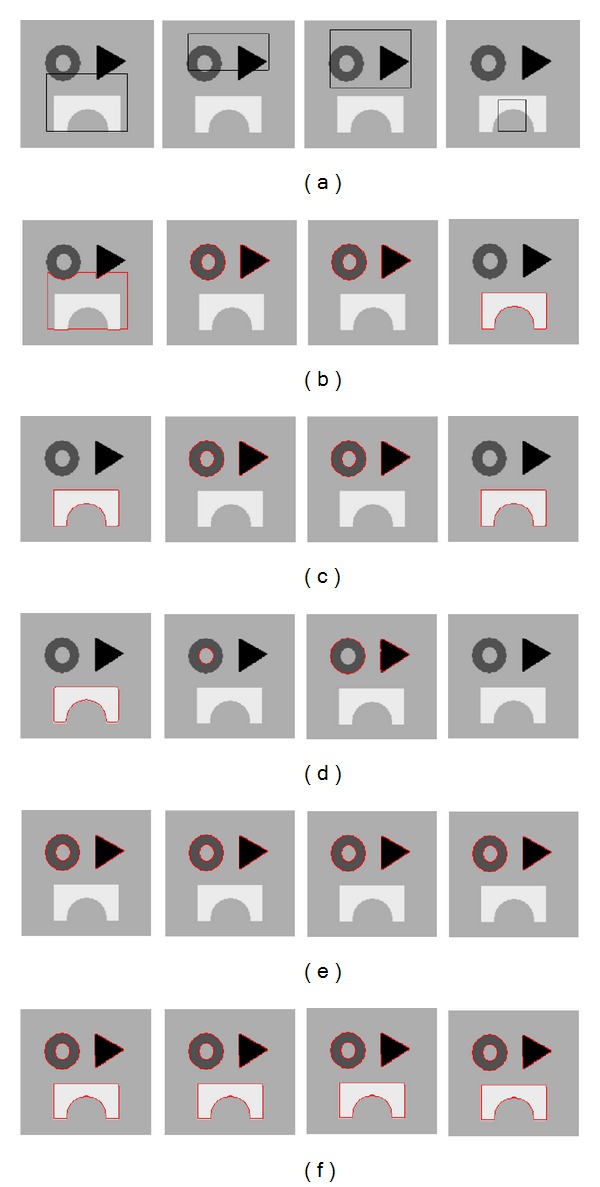
Detection of different objects from a synthetic image with various initial conditions: (a) the four different initial contours, (b) the CV model results of the corresponding initial conditions, (c) the GACV model results of the corresponding initial conditions, (d) the DRLSE model results of the corresponding initial conditions, (e) the FAC model results of the corresponding initial conditions, and (f) the LFAC model results of the corresponding initial conditions.

The effect of different initial conditions on the performance of the five models is investigated in this part. Different initial conditions are depicted in Figure 17(a). Segmentation results obtained by the five models are shown in Figures 17(b)–17(f), respectively. Segmentation results obtained by the CV model, the GACV model, and the DRLSE model are sensitive to different initial conditions. All the results obtained by the FAC model and the proposed model are the same as shown in Figures 17(e) and 17(f), which demonstrates the robustness of the two fuzzy active contour models. Moreover, compared with other four models, the proposed model can obtain the correct segmentation results under different initial conditions.

## 5. Concluding Remarks

In this paper, an enhanced partition-based fuzzy active contour model with incorporating local information for image segmentation is proposed. In the proposed model, the prototypes are not the average intensities of pixels inside and outside of the curve. Instead, they are spatially varying and are related to each pixel. By considering local image characteristics, the proposed model can efficiently segment images with intensity inhomogeneity. Moreover, to confine the update area at each iteration, shadowed sets theory is employed to adaptively detect the regions near contour boundaries, which enables the curve to evolve gradually and saves the computational resource.

The proposed approach is basically built on fuzzy active contour model with incorporating local information. The main advantages of the proposed model can be concluded as (1) computational simplicity (the calculation of each step only includes the computation of pixels within the interesting region); (2) flexibility (it has less sensitivity to initial conditions by incorporating fuzzy technique); (3) it can efficiently segment images with intensity inhomogeneity by considering image local characteristics. However, classical ACMs do not consider any spatial information in image context, which makes them sensitive to noise and other imaging artifacts to some extent. Recently, many researchers have incorporated local spatial information into segmentation algorithms to improve the performance of image segmentation. In the further work, we want to design a fuzzy active contour model which retains more image details and preserves robustness to noise by taking both spatial and gray constraints into consideration.

## Figures and Tables

**Table 1:** Quantitative result given by different models.

	CV	GACV	DRLSE	FAC	The proposed model
*P*_mp_ on Figure 6(a)	38.77%	41.91%	3.39%	38.77%	**0**
*P*_mp_ on Figure 7(a)	44.21%	48.85%	16.96%	42.66%	**9.67%**
*P*_mp_ on Figure 8(a)	38.77%	39.07%	4.57%	38.77%	**0.24%**
*P*_mp_ on Figure 9(a)	39.15%	41.54%	5.25%	39.15%	**0.32%**

## Appendix

The proofs of the center and energy differences are presented in this part. The spatial varying centers of each pixel are written in the following forms:

(A.1) $$\begin{array}{c} c_{1}\left(x,y\right)=\frac{\sum_{\Omega }{\left[u\left(i,j\right)\right]}^{m}g_{k}I\left(i,j\right)}{\sum_{\Omega }{\left[u\left(i,j\right)\right]}^{m}g_{k}},\\ c_{2}\left(x,y\right)=\frac{\sum_{\Omega }{\left[1-u\left(i,j\right)\right]}^{m}g_{k}I\left(i,j\right)}{\sum_{\Omega }{\left[1-u\left(i,j\right)\right]}^{m}g_{k}}, \end{array}$$

where (*x*, *y*) is the spatial coordinate of the current pixel. (*i*, *j*) is the pixel in the set of neighbors falling into the local region around the current pixel. *g*_*k*_ is the kernel function, which can be taken as the weight assignment of the pixel within the neighbor of the current point. *u*(*i*, *j*) is the degree of membership of pixel (*i*, *j*) belonging to the inside of the curve *C*. *m* is the fuzzy coefficient.

Assume that the gray value of the pixel is *I*_*o*_. *u*_*o*_ is the degree of membership of the pixel. The new degree of membership of the pixel is *u*_*n*_. The spatial varying centers of the pixel ${\tilde{c}}_{1}(x,y)$ and ${\tilde{c}}_{2}(x,y)$ will have a corresponding change. Considering that the membership value of only one pixel is changed, then ${\tilde{c}}_{1}(x,y)$ is given as follows:

(A.2) c~1(x,y)=∑Ω[u~(i,j)]mgkI(i,j)∑Ω[u~(i,j)]mgk=∑Ω[u(i,j)]mgkI(i,j)+unmIo−uomIo∑Ω[u(i,j)]mgk+unm−uom=s1c1(x,y)+Io(unm−uom)s1+unm−uom=(c1(x,y)(s1+unm−uom)−c1(x,y)(unm−uom)  +Io(unm−uom))(s1+unm−uom)−1=c1(x,y)+unm−uoms1+unm−uom(Io−c1(x,y)),

where *s*_1_ = ∑_*Ω*_[*u*(*i*,*j*)]^*m*^ *g*_*k*_.

The ${\tilde{c}}_{2}(x,y)$ will be given as the similar way:

(A.3) c~2(x,y)=c2(x,y)+(1−un)m−(1−uo)ms2+(1−un)m−(1−uo)m(Io−c2(x,y)),

where *s*_2_ = ∑_*Ω*_[1−*u*(*i*,*j*)]^*m*^ *g*_*k*_. Thus, the calculation of the new spatial varying centers can be easily achieved according to (A.2) and (A.3), respectively.

Besides, the change of the pixel in terms of the membership value and the spatial varying centers will cause a change in fuzzy energy. Considering that the membership value and the spatial varying centers of only one pixel are changed, the new fuzzy energy is given as follows:

(A.4) F~=∑Ω[u~(x,y)]m(I(x,y)−c~1(x,y))2︸A~ +∑Ω[1−u~(x,y)]m(I(x,y)−c~2(x,y))2︸B~.

$\tilde{A}$ and $\tilde{B}$ can be formulated separately as follows:

(A.5) A~=∑Ω[u~(x,y)]m(I(x,y)−c~1(x,y))2=∑Ω[u(x,y)]m(I(x,y)−c1(x,y))2 +unm(Io−c~1(x,y))2−uom(Io−c1(x,y))2=A+unm ×(Io−c1(x,y)−unm−uoms1+unm−uom(Io−c1(x,y)))2 −uom(Io−c1(x,y))2=A+unm(s1s1+unm−uom(Io−c1(x,y)))2 −uom(Io−c1(x,y))2=A+(unm(s1s1+unm−uom)2−uom) ×(Io−c1(x,y))2,

where *A* = ∑_*Ω*_[*u*(*x*,*y*)]^*m*^(*I*_*o*_−*c*_1_(*x*,*y*))^2^.

In a similar way, it can be proven that

(A.6) B~=B+((1−un)m(s2s2+(1−un)m−(1−uo)m)2    −(1−uo)m)(Io−c2(x,y))2,

where *B* = ∑_*Ω*_[1−*u*(*x*,*y*)]^*m*^(*I*_*o*_−*c*_2_(*x*,*y*))^2^.

As $\tilde{F}=\underset{\tilde{A}}{\underset{︸}{\sum_{\Omega }^{}{\left[\tilde{u}\left(x,y\right)\right]}^{m}{\left(I\left(x,y\right)-{\tilde{c}}_{1}\left(x,y\right)\right)}^{2}}}+\underset{\tilde{B}}{\underset{︸}{\sum_{\Omega }{\left[1-\tilde{u}\left(x,y\right)\right]}^{m}{\left(I\left(x,y\right)-{\tilde{c}}_{2}\left(x,y\right)\right)}^{2}}}$, the new energy is obtained by combining (A.5) and (A.6), which will be given as

(A.7) F~=∑Ω[u(x,y)]m(Io−c1(x,y))2+∑Ω[1−u(x,y)]m ×(Io−c2(x,y))2+(unm(s1s1+unm−uom)2−uom) ×(Io−c1(x,y))2 +((1−un)m   ×(s2s2+(1−un)m−(1−uo)m)2−(1−uo)m) ×(Io−c2(x,y))2=F+(unm(s1s1+unm−uom)2−uom)(Io−c1(x,y))2 +((1−un)m(s2s2+(1−un)m−(1−uo)m)2   −(1−uo)m)(Io−c2(x,y))2.
